# Transcriptomic analysis of wound xylem formation in *Pinus canariensis*

**DOI:** 10.1186/s12870-017-1183-3

**Published:** 2017-12-04

**Authors:** V. Chano, C. Collada, A. Soto

**Affiliations:** 1 G.I. Genética, Fisiología e Historia Forestal. ETSI Montes, Forestal y del Medio Natural. Dpto. Sistemas y Recursos Naturales, Universidad Politécnica de Madrid, Ciudad Universitaria s, /n 28040 Madrid, Spain; 2 Unidad Mixta de Genómica y Ecofisiología Forestal INIA/UPM, Madrid, Spain

**Keywords:** Wound, Healing, Conifers, Transcriptome, Wood, *Pinus canariensis*

## Abstract

**Background:**

Woody plants, especially trees, usually must face several injuries caused by different agents during their lives. Healing of injuries in stem and branches, affecting the vascular cambium and xylem can take several years. In conifers, healing takes place mainly from the remaining vascular cambium in the margin of the wound. The woundwood formed in conifers during healing usually presents malformed and disordered tracheids as well as abundant traumatic resin ducts. These characteristics affect its functionality as water conductor and its technological properties.

**Results:**

In this work we analyze for the first time the transcriptomic basis of the formation of traumatic wood in conifers, and reveal some differences with normal early- and late-wood. Microarray analysis of the differentiating traumatic wood, confirmed by quantitative RT-PCR, has revealed alterations in the transcription profile of up to 1408 genes during the first period of healing. We have grouped these genes in twelve clusters, according to their transcription profiles, and have distinguished accordingly two main phases during this first healing.

**Conclusions:**

Wounding induces a complete rearrangement of the transcriptional program in the cambial zone close to the injuries. At the first instance, radial growth is stopped, and a complete set of defensive genes, mostly related to biotic stress, are induced. Later on, cambial activity is restored in the lateral borders of the wound, even at a high rate. During this second stage certain genes related to early-wood formation, including genes involved in cell wall formation and transcription factors, are significantly overexpressed, while certain late-wood related genes are repressed. Additionally, significant alterations in the transcription profile of abundant non annotated genes are reported.

**Electronic supplementary material:**

The online version of this article (10.1186/s12870-017-1183-3) contains supplementary material, which is available to authorized users.

## Background

Organisms usually suffer injuries throughout their life. In multicellular organisms these injuries can cause the damage or loss of differentiated tissues or organs, and ease the entry and spread of pathogens. The analysis of the similarities and differences in the regeneration process in animals and plants has been on the spotlight in recent years [1–3]. Animals can often regenerate these damaged tissues and even, in certain cases, the lost organs, and due to constant regeneration of certain tissues such as skin, eventually no signal of the injury remains after some time.

On their side, plants do not regenerate continuously their tissues. Proliferation is usually limited to certain niches: the root and shoot apical meristems (including axillary buds) and the vascular cambium and the phellogen in woody plants. If damage occurs, the plant generates new tissues and organs from these meristems or eventually develops new meristematic niches from living cells, usually parenchymatic ones [4, 5]. This is the case of the traumatic periderms developed, for instance, from cortical parenchyma or from mesophyll to seal a wound in a young stem or a leave.

When a woody branch or stem suffers a deep wound, affecting the secondary xylem, the vascular cambium must be restored. In certain angiosperms proliferation from xylem parenchyma or from immature xylem conducting elements has been described, as in *Tilia* [6], *Eucommia* [7] or *Populus* [8]. These cells can reverse their differentiation pathway and divide profusely, giving rise to a parenchymatic callus. Later on, a new, traumatic vascular cambium differentiates within this callus, and new secondary xylem and phloem are produced.

On the contrary, this proliferation from (partially) differentiated cells is not usual in conifers. In these species healing takes place mainly from the remaining vascular cambium in the margins of the wound, as described recently for *Pinus canariensis* [9].

Anyway, the traumatic wood formed this way can be easily distinguished from normal wood. Traumatic wood usually presents malformed tracheary elements and fibers, with altered lignification patterns, and with a high proportion of parenchymatic cells. Orientation of these elements is also very often distorted [9–12], probably due to altered hormonal flux, and also to altered mechanical signals, as suggested by Chano *et al*. [9]. This disorganized xylem implies an evident disadvantage for water and nutrient transport [10]. In the case of conifers, especially Pinaceae, traumatic wood also presents a very high proportion of resin ducts, as described for *Cedrus libani* [13], *Larix decidua* [14, 15], *Picea abies* [15, 16], *Pinus nigra* [17] or *Pinus pinaster* [18]. Actually, formation of traumatic resin ducts is the basis of traditional resin exploitation, very common in the past for several species of Mediterranean pines, and with increasing interest in the last years [18].

Since plants do not renew their secondary xylem, but generate new sheets of xylem, centrifugally, year after year, traumatic xylem also remains in the damaged branch or stem, leaving a “scar” in the wood. These scars have proven to be very useful, for instance, for dendrochronology studies [15, 19]. However, traumatic wood presents undesirable characteristics from a technological point of view. Although the higher density due to the increase in resin content can improve certain mechanical qualities of wood it also causes problems at machining and blunting [20]. In addition, disordered and not properly formed traumatic tracheids contribute to alter the physico-mechanical properties of wood. Therefore, lumber dealers consider traumatic wood as a defect, lowering the price and reducing the applicability of wood pieces with important scars.

Several works have focused in the consequences of traumatisms on wood development in conifers: early-late wood ratio, ring width, formation of traumatic resin ducts (f.i., [10, 21–24]), and a few others in the description of the healing process from an anatomical point of view [9, 25–27]. However, although the molecular aspects of the response to traumatism has been analysed in different angiosperms (f. i., [4, 28–31]), the process has been less studied in gymnosperms, where most works have focused in the induction of traumatic resin ducts by traumatism, insect attack or fungal infections [32–35]. In this work we focus on the molecular basis of traumatic wood formation in a gymnosperm, *P. canariensis*, known for its extraordinary healing ability. For this purpose we have performed deep wounds in the stem of *P. canariensis* trees, affecting the vascular cambium, and have assessed the transcriptomic profile during the healing process and traumatic wood growth.

## Results and discussion

### Identification of genes induced and repressed in response to wounding

In order to analyse the transcriptomic response in the cambial zone and differentiating xylem in the borders of deep wounds performed in the stem of pine trees, we hybridized a 60K two-color cDNA microarray (Agilent, USA) which includes genes involved in *P. canariensis* xylogenesis [36]. Samples were collected at three dates during wound response: i) H1 was collected seven days after wounding, ii) H2 after 75 days, when development of traumatic wood was evident and while the trees outside the wound area were still forming early wood, and finally iii) H3 92 days after wounding, when the trees were already forming late wood. Controls for each sample were collected at the same sampling dates from branches distant from the wound, in order to distinguish local effects caused by wound response from constitutive changes in gene expression during the vegetative season.

**Fig. 1:**
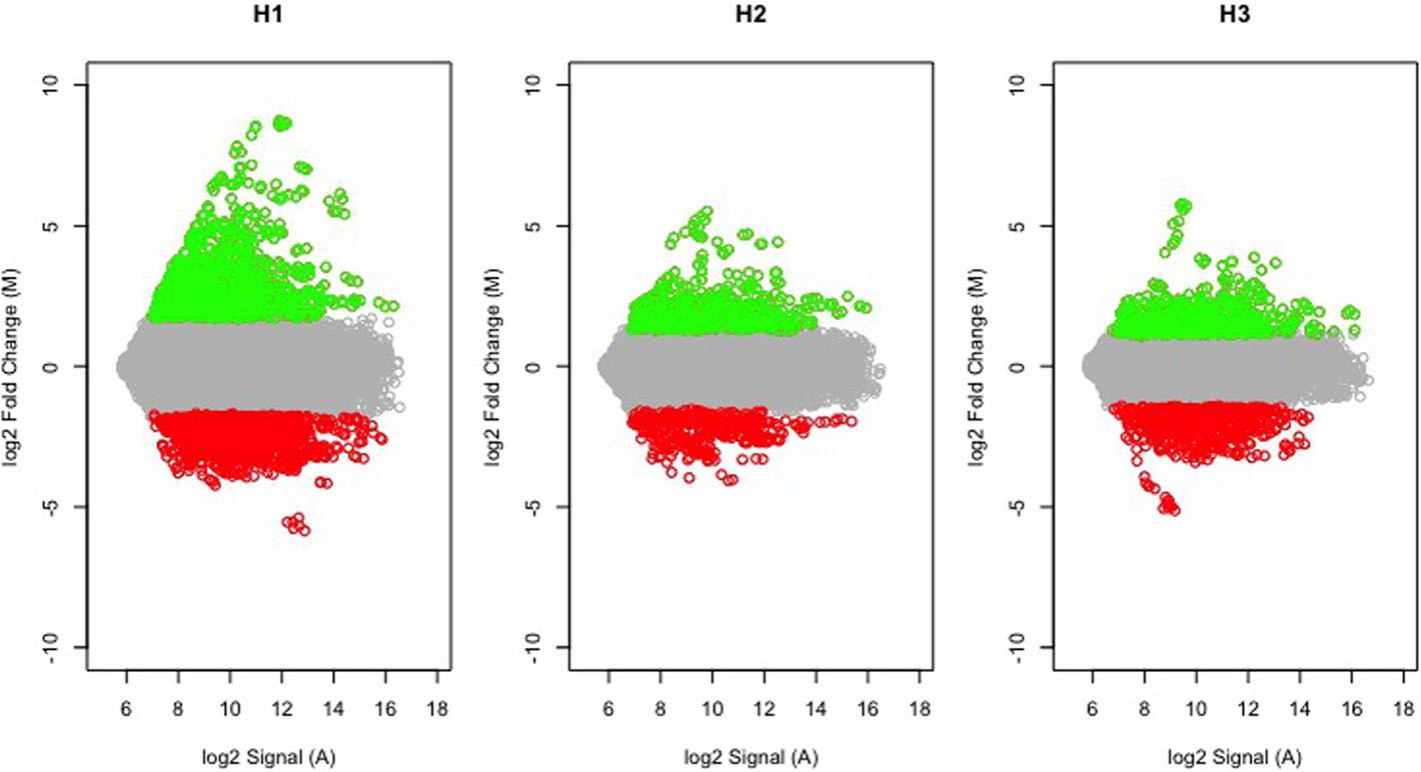
MA plot of microarray normalized data during wound-response. X-axis: Log2 of microarray signals; Y-axis: Log2 of Fold Change values; Green dots: probes selected as overexpressed (FC > 2, FDR < 0.05, between treatment and control RNA samples); Red dots: probes selected as underexpressed (FC < -2, FDR < 0.05 between treatment and control RNA samples)

Figure 1 shows the distribution of genes selected as over- and underexpressed at each sampling point. We identified 1408 differentially expressed genes (DEG), genes significantly overexpressed or repressed compared to normal wood formation. Table 1 shows a selection of 91 DEGs with the strongest response (induction or repression), grouped following the functional processes they are presumably related to, according to their top BLASTx hit, as previously described [36]. The complete table can be found in a supplementary table (Additional file 1).

**Table 1:** Selected wound-responsive DEGs, grouped according to their putative role and their expression profile clustering

Cluster	ID	Seq. Description	H1		H2		H3	
			FC	FDR	FC	FDR	FC	FDR
Defense and stress response genes								
A	Contig18804	disease resistance response protein 206-like	**395.78**	**0.00**	**37.14**	**0.00**	**52.52**	**0.00**
A	Contig19053	pathogenesis-related protein pr-4b-like	**96.55**	**0.00**	**23.91**	**0.00**	**19.77**	**0.00**
A	Contig22185	major allergen pru ar 1-like	**138.29**	**0.00**	**13.75**	**0.00**	**4.94**	**0.01**
A	Contig22375	pathogenesis-related protein pr-4-like	**343.24**	**0.00**	**7.69**	**0.00**	**7.38**	**0.00**
A	Ppnisotig12265	antimicrobial peptide 1-like	**72.52**	**0.00**	**16.88**	**0.00**	**12.84**	**0.00**
A	Ppnisotig13133	pathogenesis-related protein pr-4-like	**206.36**	**0.00**	**6.43**	**0.00**	**5.13**	**0.00**
A	Ppnisotig13431	disease resistance response protein 206-like	**133.78**	**0.00**	**24.75**	**0.00**	**31.68**	**0.00**
B	Contig00602	defensin ec-amp-d2-like	**11.27**	**0.00**	2.07	0.12	-1.42	0.65
B	Contig02906	nematode resistance hspro2-like	**7.81**	**0.01**	-1.23	0.94	-2.12	0.11
B	Contig03079	lactoylglutathione lyase glyoxalase i family protein	**3.96**	**0.04**	1.97	0.20	-1.36	0.75
B	Contig09180	thioredoxin h-type	**6.82**	**0.01**	1.52	0.44	1.39	0.55
B	Contig19857	nematode resistance hspro2-like	**9.88**	**0.00**	-1.33	0.83	-2.56	0.06
B	Contig20555	phenylalanine ammonia-lyase-like	**14.41**	**0.00**	**3.19**	**0.03**	-1.08	1.11
C	Contig13499	(-)-camphene tricyclene chloroplastic-like	2.17	0.17	2.14	0.12	**2.81**	**0.01**
D	Contig00126	basic endochitinase a-like	**15.25**	**0.00**	-1.20	0.55	0.34	1.13
D	Contig10307	endochitinase a-like	**65.05**	**0.00**	**3.40**	**0.02**	1.49	0.47
D	Contig17617	defensin ec-amp-d2-like	**44.89**	**0.00**	1.32	0.40	-1.23	0.69
D	Contig21216	endochitinase a-like	**92.45**	**0.00**	2.28	0.08	**2.51**	**0.02**
D	Contig23442	chitinase 1-like	**29.31**	**0.00**	2.12	0.37	-1.65	0.41
D	Ppnisotig00751	endochitinase pr4-like	**85.39**	**0.00**	**3.16**	**0.02**	1.28	0.78
D	Ppnisotig01747	peroxidase 12-like	**97.76**	**0.00**	1.74	0.28	1.53	0.37
D	Ppnisotig06171	glutathione s-transferase f9-like	**41.49**	**0.00**	**2.48**	**0.05**	1.55	0.36
D	Ppnisotig08058	endochitinase pr4-like	**89.60**	**0.00**	1.15	1.04	1.12	1.08
H	Contig03270	geranylgeranyl pyrophosphate chloroplastic-like	1.61	0.46	1.48	0.45	**2.57**	**0.03**
H	Contig08417	abietadienol abietadienal oxidase-like	1.10	1.12	2.08	0.11	**2.64**	**0.02**
H	Ppnisotig10634	geranylgeranyl pyrophosphate chloroplastic-like	-1.26	1.00	**2.54**	**0.04**	**2.92**	**0.01**
Cell-wall matrix development and/or carbohydrates metabolism								
G	Contig06476	caffeoyl- o-methyltransferase	-1.27	0.97	**-3.59**	**0.04**	-1.44	0.61
G	Contig15857	cellulose synthase-like protein d3	1.38	0.78	-1.14	1.08	**-3.00**	**0.04**
G	Contig17013	probable xyloglucan endotransglucosylase hydrolase protein 23	-1.24	0.93	**-6.29**	**0.00**	**-7.64**	**0.00**
G	Contig21865	galactinol--sucrose galactosyltransferase-like	2.27	0.18	-2.03	0.21	**-3.39**	**0.03**
H	Contig00603	beta-xylosidase alpha-l-arabinofuranosidase 2-like	-0.57	1.10	2.46	0.07	**2.58**	**0.03**
H	Contig03225	expansin alpha	-2.10	0.20	**2.64**	**0.04**	1.99	0.13
H	Contig05066	probable pectate lyase 15-like	**-5.21**	**0.01**	2.08	0.20	**3.24**	**0.01**
H	Contig05424	probable xyloglucan endotransglucosylase hydrolase protein 8-like	-1.78	0.37	**2.55**	**0.04**	2.18	0.06
H	Contig09907	probable xyloglucan endotransglucosylase hydrolase protein 32	-1.86	0.32	2.14	0.09	**2.95**	**0.01**
H	Contig10173	endoglucanase 24-like	**-3.26**	**0.05**	1.58	0.46	**2.58**	**0.03**
H	Contig13281	probable pectinesterase 68-like	-1.56	0.60	2.18	0.09	**3.40**	**0.01**
H	Contig18777	endoglucanase 24-like	-1.69	0.41	**2.54**	**0.04**	**5.32**	**0.00**
H	Contig18811	expansin alpha	-1.59	0.58	**3.42**	**0.01**	**4.98**	**0.00**
I	Contig00766	xyloglucan endotransglucosylase hydrolase protein 9-like	**-17.49**	**0.00**	-1.36	0.72	-1.24	0.78
I	Contig02447	caffeoyl- o-methyltransferase-like	**-6.41**	**0.01**	1.17	0.75	-1.58	0.34
I	Contig03231	probable carboxylesterase 15-like	**-4.22**	**0.03**	-1.63	0.38	-1.39	0.68
I	Contig08356	udp-glycosyltransferase 85a2-like	**-44.90**	**0.00**	1.62	0.28	1.45	0.46
I	Contig11436	probable polygalacturonase non-catalytic subunit jp650-like	**-13.41**	**0.00**	1.46	0.47	1.52	0.44
I	Contig13611	beta-xylosidase alpha-l-arabinofuranosidase 2-like	**-4.92**	**0.02**	0.35	1.08	1.39	0.56
I	Contig19457	xyloglucan endotransglucosylase hydrolase protein 9-like	**-14.61**	**0.00**	-1.41	0.66	-1.31	0.68
L	Contig00654	cellulose synthase a catalytic subunit 3	**-8.70**	**0.00**	-1.86	0.26	-1.57	0.54
L	Contig01405	protein cobra-like	**-6.74**	**0.01**	-2.06	0.20	-2.27	0.09
L	Contig01916	xyloglucan glycosyltransferase 4-like	-1.45	0.71	-1.36	0.60	**-4.36**	**0.01**
Hormone signalling								
B	Contig00715	1-aminocyclopropane-1-carboxylate oxidase-like	**5.91**	**0.01**	1.63	0.51	1.77	0.19
B	Contig03482	salicylic acid-binding protein 2-like	**5.92**	**0.01**	1.99	0.14	1.40	0.57
C	Contig00524	1-aminocyclopropane-1-carboxylate oxidase-like	1.69	0.38	**3.80**	**0.02**	**2.65**	**0.02**
C	Ppnisotig12073	1-aminocyclopropane-1-carboxylate oxidase-like isoform x1	**3.27**	**0.05**	**2.48**	**0.05**	**2.29**	**0.04**
D	Contig14053	salicylic acid-binding protein 2-like	**22.28**	**0.00**	**3.04**	**0.04**	**3.76**	**0.00**
I	Contig16100	1-aminocyclopropane-1-carboxylate oxidase	**-4.81**	**0.02**	**2.54**	**0.04**	**2.45**	**0.04**
Transcriptional regulation of meristem activity								
B	Contig00787	nac domain-containing protein 2-like	**9.26**	**0.00**	-1.02	0.85	-1.26	0.79
B	Contig12353	early nodulin-like protein 1	**9.58**	**0.00**	-1.48	0.61	**-2.80**	**0.05**
B	Contig20304	homeobox-leucine zipper protein athb-13-like	**3.82**	**0.04**	1.31	0.72	1.24	0.86
D	Contig13895	nac transcription factor 29-like	**22.81**	**0.00**	1.08	1.07	1.20	0.64
G	Contig09007	exordium like 2	-0.36	1.17	**-3.66**	**0.03**	**-4.59**	**0.01**
H	Contig06813	wuschel-related homeobox 4-like	-1.76	0.34	1.49	0.47	**2.61**	**0.02**
H	Contig14178	clavata3 esr-related 12 family protein	**-3.46**	**0.05**	1.70	0.41	**2.45**	**0.03**
L	Contig05551	probable wrky transcription factor 51-like	-2.28	0.19	**-3.39**	**0.04**	-2.23	0.15
L	Contig12050	transcription factor myb46-like	**-9.05**	**0.00**	-2.05	0.18	-2.11	0.22
Non annotated genes and unknown functions								
A	Contig03506	hypothetical protein SELMODRAFT_115352	**63.88**	**0.00**	**20.85**	**0.00**	**12.74**	**0.00**
B	Contig09209	---NA---	**13.90**	**0.00**	1.35	0.82	-1.67	0.36
B	Contig22448	---NA---	**12.00**	**0.00**	1.31	0.68	-1.40	0.64
B	Contig24621	---NA---	**12.34**	**0.00**	0.36	1.01	-1.81	0.30
D	Contig19474	---NA---	**22.81**	**0.00**	1.49	0.64	0.36	1.09
D	Contig20761	predicted protein	**24.06**	**0.00**	1.29	0.75	1.80	0.20
D	Contig22397	---NA---	**31.25**	**0.00**	2.29	0.09	1.10	1.10
D	Contig23569	---NA---	**36.23**	**0.00**	2.55	0.06	-2.05	0.27
F	Contig03012	---NA---	**7.04**	**0.01**	**-7.98**	**0.00**	**-4.60**	**0.01**
F	Contig03111	---NA---	**5.57**	**0.02**	-3.12	0.12	**-2.77**	**0.05**
G	Contig12685	uncharacterized loc101213469	1.34	0.82	-1.79	0.31	**-6.43**	**0.00**
G	Contig20076	---NA---	2.71	0.12	0.11	1.03	**-7.76**	**0.00**
G	Contig23934	---NA---	1.31	0.79	-2.74	0.08	**-9.51**	**0.00**
G	Contig24690	---NA---	1.55	0.53	-2.30	0.17	**-8.11**	**0.00**
H	Contig12627	---NA---	-1.94	0.28	**4.42**	**0.00**	**8.62**	**0.00**
H	Contig21346	---NA---	-1.02	1.19	**7.69**	**0.00**	**7.90**	**0.00**
I	Contig02729	---NA---	**-11.85**	**0.00**	2.03	0.19	1.70	0.23
I	Contig13781	---NA---	**-12.25**	**0.00**	-1.08	1.01	1.09	0.88
I	Contig16419	---NA---	**-13.45**	**0.00**	1.55	0.31	-0.45	0.83
I	Contig19504	---NA---	**-12.31**	**0.00**	-1.05	1.03	1.13	0.90
L	Contig02798	uncharacterized loc101210414	-1.25	0.99	-1.78	0.29	**-5.06**	**0.01**
L	Contig10360	---NA---	**-9.23**	**0.00**	-2.42	0.12	-1.69	0.36
L	Contig12514	---NA---	**-9.36**	**0.00**	-2.14	0.17	-1.35	0.33
L	Contig14134	---NA---	-1.64	0.55	**-9.50**	**0.00**	**-3.12**	**0.04**
L	Contig14477	---NA---	**-10.08**	**0.00**	-2.38	0.12	-2.15	0.12
L	Contig20478	---NA---	-1.33	0.86	**-5.43**	**0.01**	**-2.76**	**0.04**
L	Contig34794	PREDICTED: uncharacterized protein LOC101509257	**-10.94**	**0.00**	**-3.20**	**0.04**	-2.59	0.10

FC: Fold-change. FDR: adjusted p-value by False Discovery Rate. In bold, statistically significant values

**Fig. 2:**
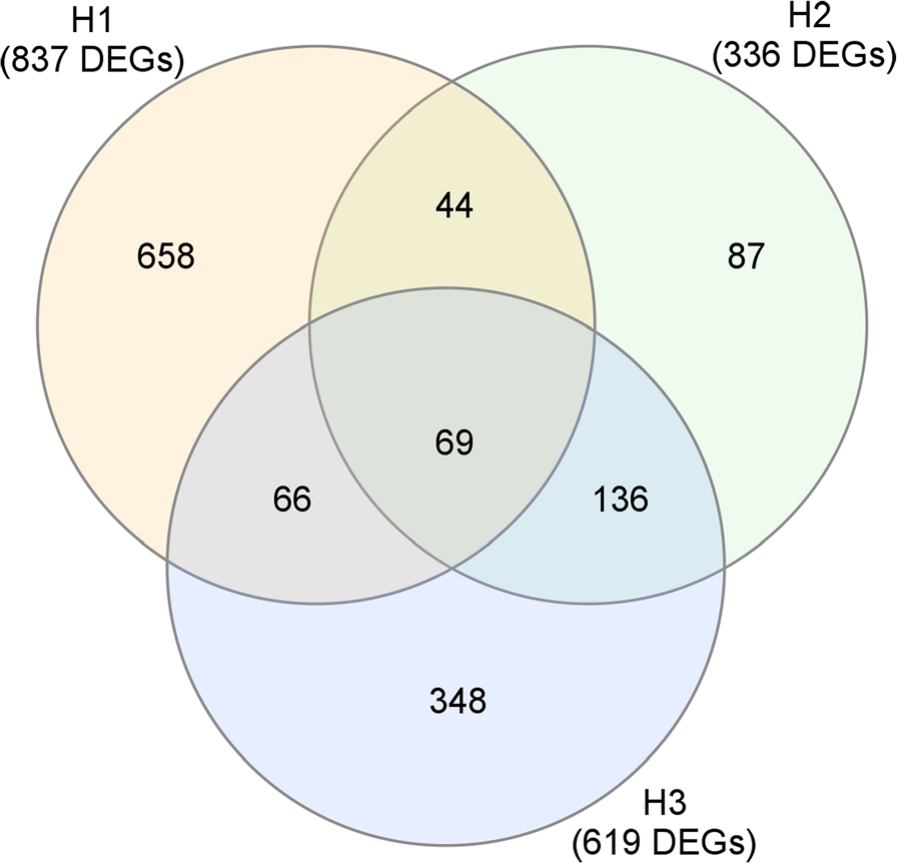
Differentially expressed genes during first healing. Venn’s diagram of wound-responsive DEGs at 7 (H1), 78 (H2) and 92 (H3) days after wounding

Immediate response H1 included 837 DEGs; 619 DEGs were detected for H3, while only 336 DEGs were detected at H2. Just 69 genes were identified as DEG for the three sampling dates, H1, H2 and H3. Moreover, just 87 genes were identified as DEGs exclusively for H2, while 348 were exclusive DEGs for H3 and up to 658 for H1 (Fig. 2).

Enrichment analysis of DEGs pointed out an increase of mRNA levels for the categories “defense response”, “response to stress” and different forms of “response to stimulus”, as “response to abiotic stimulus” or “response to biotic stimulus”, among others, in the Biological Process (BP) category. As well, other enriched GO terms were “nucleic acid binding transcription factor activity” and “sequence-specific DNA binding transcription factor activity”, for the Molecular Function (MF) category, and “extracellular region”, “cell wall”, “cell periphery” and “external encapsulating structure” GO terms of the Cellular Component category.

### Hierarchical clustering of DEGs

**Fig. 3:**
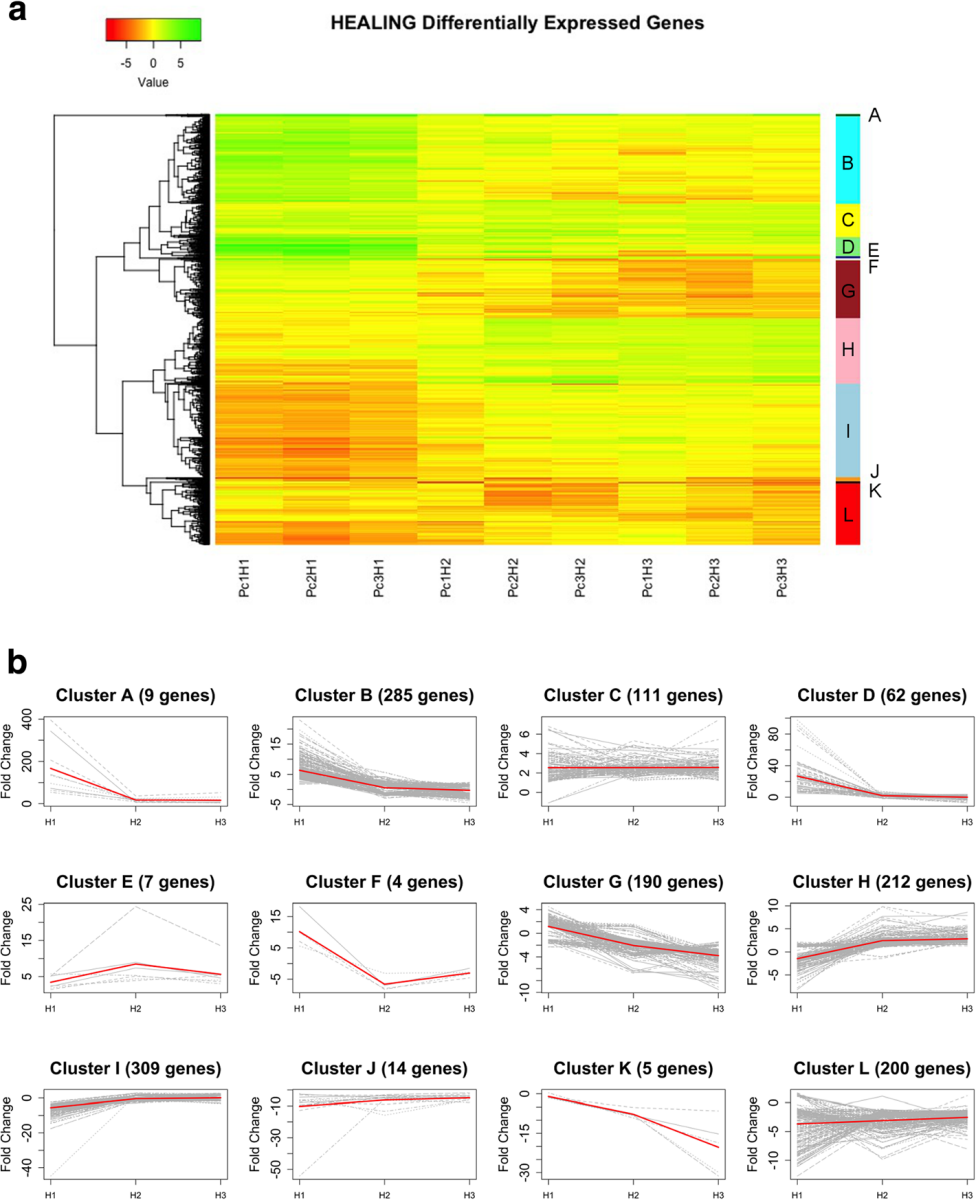
Clustering of DEGs according to expression patterns. **a** Hierarchical clustering of 1408 DEGs for three biological replicates (PC1, -2 and -3), identifying 12 clusters (A – L). **b** Gene expression profiling of clusters, showing Fold Change variations along sampling dates

Twelve clusters were established according to the transcription patterns detected for DEGs throughout H1, H2 and H3 (Fig. 3).

Cluster A includes genes clearly induced at H1, which keep high transcription levels during H2 and H3, although at a minor degree. Genes included in clusters B, D and F were also overexpressed at H1, but later on their transcription levels decrease, being even repressed at H2 and/or H3. Genes in cluster C show a faint overexpression throughout the three phases, while clusters E and H show an increasing overexpression at H2 and H3. The opposite pattern is reflected by clusters G and K, with an increasing repression at H2 and H3. Genes in cluster I show a significant repression at H1, followed by a recovery of transcription to normal levels at H2 and H3. Finally, clusters J and L are characterized by a general repression during the three phases.

Clustering of samples revealed consistency among biological replicates, as shown in a supplementary figure (Additional file 2). Samples harvested at H1 clustered together and separated from the other sampling dates; on their side, H2 and H3 samples were included in the same group. Slight irregularities (for instance, sample Pc3H2 is closer to H3 samples than to the other H2 ones) can be due to the genetic variability among trees. This result supports the differentiation of two major phases (H1 and H2/H3) in the response to wounding, as discussed below.

To validate the reliability of the transcription profiles obtained from microarray hybridization, we selected 12 genes for qRT-PCR analysis, covering the main tendencies described above and the putative function of the genes. Thus, we selected three genes directly involved in cell growth and cell wall formation, as an expansin (Contig 03225, cluster H), a CeSA-like (Contig 00654, cluster L) and a CCoAOMT (Contig 06476, cluster G), transcription factors also involved in xylogenesis, as a MYB46-like (Contig 12050, cluster L), a WOX4-like (Contig 06813, cluster H), a bHLH35-like (Contig 05923, cluster C)an ATHB13-like (Contig 20304, cluster B), a NAC2-like (Contig 00787, cluster B), and a WRKY51-like (Contig 05551, cluster L). Finally, we also analysed a gene coding for a PAL protein (Contig 20555, cluster B), involved in salicylic acid biosynthesis and presumably related to defense, an EXORDIUM-like protein (Contig 09007, cluster G), presumably involved in cell proliferation, and a Major Allergen Pru AR1-like (Contig 22185, cluster A), putatively involved in defensive response.

**Fig. 4:**
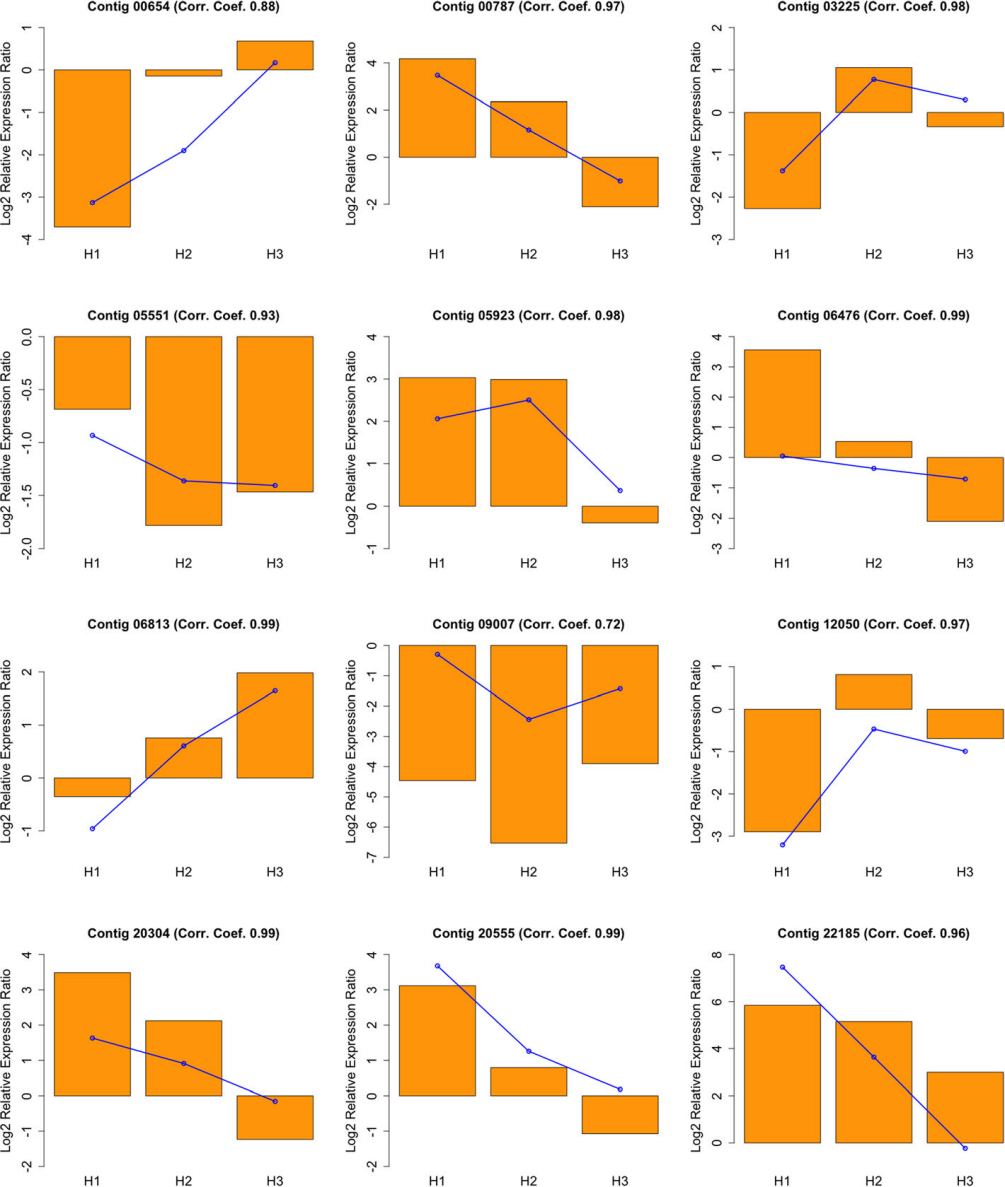
qRT-PCR validation of microarray transcription profiles. X-axis: sampled times; Y-axis: normalized gene expression values of selected DEGs for qRT-PCR (bars) validation of microarray expression profiling (continuous lines)

Profiles obtained by qRT-PCR for these genes match the ones obtained from microarray hybridization, with high correlation coefficients, thus validating the general tendencies described above for microarray analysis (Fig. 4).

Detailed analysis of the transcription patterns of the differentially expressed genes (DEGs) leads to the identification of two major phases in the response to wounding.

#### H1. Immediate response

A complete rearrangement of the transcriptional program takes place as immediate response to wounding. At H1, a general repression of genes involved in the normal development of early wood is detected. In particular, genes related to meristematic activity, cell division or synthesis of cell wall show their transcription levels significantly lowered. This is consistent with anatomical observations: Chano *et al.* [9] described how cambial activity is stopped in response to a recent wound, and no growth in the cambial zone is further detected up to approximately 4 weeks after wounding. On the contrary, numerous genes putatively involved in the defense against stress (including biotic stress) are significantly induced at this point, serving as a defense against opportunistic pathogens infecting the wound. Noteworthy, many of these genes were reported to show their transcript maximum in normal xylogenesis during late wood formation [36]. Expression of genes related to stress and defense processes in differentiating late wood has also been reported in other species. For instance, Mishima *et al*. [37] described the abundance of “defense mechanism genes” in the “cessation of growth clusters” obtained from cambial zone and differentiating xylem in *Cryptomeria japonica* In normal late wood formation, these genes could act as a preventive defense against putative pathogens infecting the tree just before dormancy, since dormancy could hamper the display of an induced response during winter. Later on, well differentiated late wood constitutes a barrier against eventual infections that could take place during winter, as described by the CODIT (Compartmentalization Of Decay In Trees) model [38].

Thus, among the genes involved in **cell wall development** typically overexpressed during early wood development and repressed at H1 we can find **transcription factors** such as the HD-ZIP class III family member ATHB15-like or a MYB46-like transcription factor, reported to be involved in cell wall biosynthesis in *Arabidopsis* [39], included in clusters I and L, respectively (Fig. 3). Other genes directly involved in the cell wall biosynthesis and repressed at this stage, were some CAZymes (f.i., Contigs 03231, 11436, 13611, 19457, 00766, or 08356), COBRA or KORRIGAN endoglucanase (Contigs 01405, or 10173, as well as a CCoAOMT (Contig 02447), involved in lignin biosynthesis and deposition. As well, a homologous of the rice NAC29 transcription factor (Contig 13895) has been found to be locally induced at H1 (FC=22.81). This gene was previously reported to participate in normal late-wood development in the Canary Island pine [36], coexpressed with a putative cellulose synthase-like protein, consistently with its reported role as activator of CesA in rice [40]. However, no significant induction of CesA was found in H1. This fact can be due to the observed repression at this point of MYB546, another activator of CesA [39]. Additionally, NAC29 could be also involved in other routes related to wound stress, and not only in the synthesis of cellulose, since no growth was detected at H1. Actually, this gene has been described to be involved in the response to stress caused by high salinity and drought in bread wheat [41].

Conversely, among the genes significantly overexpressed at H1 we can find genes related to oxidative stress, hydrolytic enzymes and hormonal signaling. **Oxidative stress** is one of the main effects of mechanical damage and infections. Cell lysis results in the production of hydrogen peroxide, which is toxic for pathogens, but also for plant cells, triggering the hypersensitive response [42]. Peroxidases are then induced for ROS (reactive oxygen species) detoxification [43, 44]. Interestingly, in addition to their role in response to pathogens [45, 46], peroxidases are also involved in lignin biosynthesis and suberization [47–49]. Although some peroxidases were repressed at H1, several contigs coding for different isoforms of a peroxidase12-like protein were overexpressed at this time (f.i., Ppnisotig 01747 with FC value of 97.76, cluster D). In the same way, other genes involved in oxidative stress were induced at this stage, as Contig 03079 (cluster B), putatively coding for a lacoylglutathione lyase, involved in the glutathione-based detoxification [50] and in the response to drought and cold stress [51]. In the same way, a glutathione-S-transferase (Ppnisotig 06171 found in cluster D) or a thioredoxin (Contig 09180, cluster B), also involved in the anti-oxidative plant defense [52], were also overexpressed at H1.

Another important group of genes induced at this stage are those coding for **hydrolytic enzymes** that attack pathogen cell wall. Among them we find Contig 18804 and Ppnisotig 13431 in cluster A, homologous to PI206, a disease resistance response protein firstly described in *Pisum sativum*, where is induced after inoculation with *Fusarium solani* [53, 54]. In the same way, and also in cluster A, the putative PR-4-like proteins Contig 22375, Ppnisotig 13133 and Contig 19053 are highly induced at H1 (FC values of 343.24, 206.36 and 96.55, respectively). PR-4 protein was first described in *Solanum tuberosum* [55], named also win-1 and win-2 for “wound-inducible genes”. In *Capsicum chinense* L., PR-4 was found to have both RNAse and DNAse activity in the extracellular space during stress conditions [56]. Other putative PR4-like proteins with endochitinase activity [57], as Ppnisotig 08058 and Ppnisotig 00751, were also induced at H1 and found in cluster D. This cluster also includes other chitinases, such as endochitinase a-like proteins (Contig 21216, Contig 10307 and Contig 00126), and chitinase 1-like protein (Contig 23442). A major allergen pru ar1 homologous (Contig 22185) was also found in cluster A, strongly induced at H1 (FC 138.29). This protein was first described in *Prunus armeniaca* during ripening and annotated as a pathogenesis-related protein [58]. Ppnisotig 12265, found in cluster G, corresponds to a putative antimicrobial peptide 1, which are widely present in living organisms, and possess antifungal and antibacterial properties [59]. Finally, we can also mention Contig 00602 (cluster B) and Contig 17617 (cluster D), coding for two defensins, the most abundant antimicrobial peptides in plants, involved in defense-related processes, biotic stress response and plant development [60], which were also reported to be expressed during normal late wood differentiation in *P. canariensis* [36].

In the first stage after wounding the plant displays an extensive **hormonal signaling**. For instance, Contig 00715 and Ppnisotig 12073, included in clusters B and C, respectively, encode for putative 1-aminocyclopropane-1-carboxilate oxidase (ACO) proteins, involved in the synthesis of ethylene, known to be involved in different stress- and defense-related processes [61, 62].

Jasmonic acid (JA) is known to trigger a complex signaling network, both locally, activating the expression of wound-induced genes, and systemically, via the systemin peptide [63], mediated by ethylene [64]. However, we have not detected any DEG related to JA biosynthesis. The restrictive criteria used in this work to select DEGs can account for this result. Additionally, a local repression of the JA-dependent pathway by ethylene production has been reported in *Arabidopsis* [65], where the existence of an additional JA-independent pathway has also been described. This could also be the case for *Pinus canariensis.*

Two genes coding for salicylic acid-binding protein 2-like (SABP2-like) proteins were overexpressed at H1 (Contig 03482, cluster B) and at H1 and H2 (Contig 14053, cluster D). These proteins are involved in the plant immune response, through their salicylic acid (SA)-stimulated lipase activity [66]. SA is also involved in the expression of plant pathogenesis-related genes [67], and is thought to be an antagonistic of JA [68], blocking its synthesis [69, 70]. This would also be consistent with the lack of detection of DEGs related to the JA-dependent wound response pathway in this work.

Several non-annotated genes differentially overexpressed at H1 showed high levels of overexpression, specifically 63 DEGs in cluster B, 11 in cluster D, as many of the DEGs related to defense and stress mentioned above, and 2 in cluster F (f.i., Contigs 09209, 22448 or 24621 in cluster B, Contigs 23569, 22397 or 19474 in cluster D, with FC values over 20, or Contigs 03012 and 03111 in F). In addition, other non-annotated DEGs were repressed at H1, mainly grouped in clusters I (86 DEGs, with FC values below -10 for Contigs 02729, 13781, 19504 and 16419), and L (30 DEGs, with FC values close to -10 for Contigs 10360, 12514 or 14477). Additionally, other poorly annotated genes can be found among the H1-related DEGs. For instance, Contig 03506 was strongly induced at H1, with a FC value of 63.88, and kept overexpressed in H2 and H3. This sequence was annotated as homologous of the hypothetical protein SELMODRAFT_115352, predicted in the clubmoss *Selaginella moellendorfii* [71]. As well, other remarkable contigs poorly annotated were Contig 20761 (cluster D), a predicted protein with FC value of 24.06 in H1, and Contig 34794 (cluster L), repressed to -10.94 at H1 and annotated as homologous to chickpea uncharacterized protein LOC101509257 [72].

#### H2/H3: Development of traumatic wood.

Chano *et al.* [9] described that noticeable formation of traumatic xylem begins 4 weeks after wounding. Accordingly, we collected traumatic wood samples 11 weeks after wounding (H2), when traumatic growth was visible at healing borders. At this date, early wood was still being formed [36]. Two weeks later, when the trees were already differentiating late wood [36], another samples of traumatic wood were collected in independent wounds (H3).

As expected, after the first phase, characterised by the cessation of growth and by the expression of defensive genes, cambial activity resumes at the wound margins and development of traumatic wood is evident. Consistently, genes related to **cell proliferation and cell wall biosynthesis** are expressed. Thus, transcription patterns are more similar between H2 and H3, and differ more from H1.

**Fig. 5:**
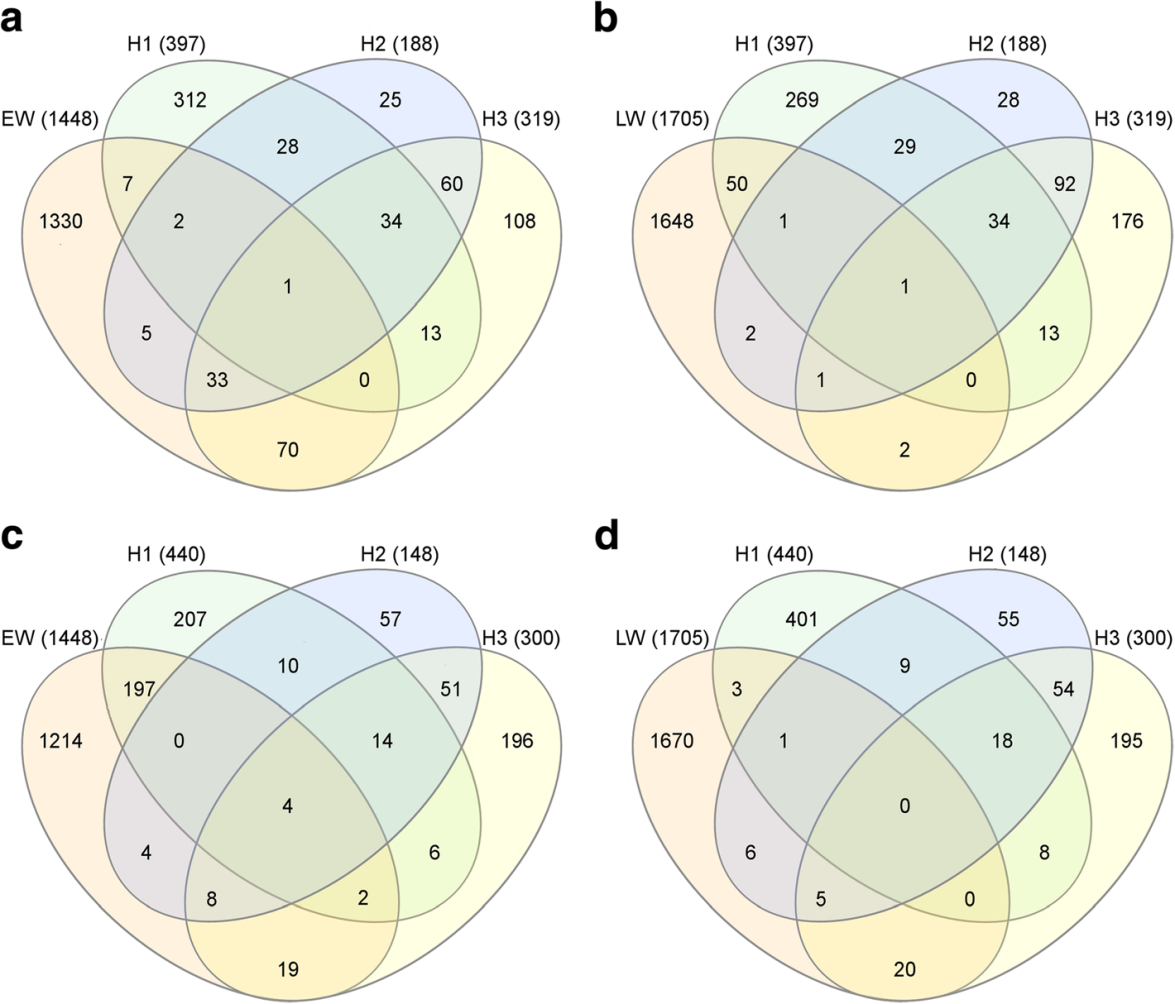
Expression of xylogenesis-related genes during wound response. Venn’s diagrams of wound-induced DEGs and early-(**a**) and late-wood (**b**) genes, and of wound-repressed DEGs and early-(**c**) and late-wood (**d**) genes

While most of the genes involved in xylogenesis during latewood formation do not change their normal transcription patterns, and therefore are not detected as DEG at H2 or H3, several genes characteristic of early wood formation appear as overexpressed at these phases. This is the case for Contig 06813 (cluster H), encoding for a WOX4-like transcription factor. WOX4 belongs to the WUSCHEL-related HOMEOBOX (WOX) family, which is involved in the differentiation in the organizing center of the apical shoot [73], in procambial and cambial growth with function in vascular bundles development [74, 75] and in the regulation of proliferation from stem cells niches in root and shoot meristems after embryogenesis [76] together with CLAVATA (CLV; [77]). Moreover, a homologous of the clavata3-like protein (CLV3) was found to be induced at H3 as well (Contig 14178, cluster H), suggesting similar combined roles in response to wounding and meristematic activity during tissue regeneration and traumatic wood development. In the same way, homologues of two expansins (Contigs 03225 and 18811), two KORRIGAN endoglucanases (f.i., Contigs 18777 and 10173) or several CAZymes (f.i., Contigs 00603, 13281, 09907, 05424, or 05066), typically expressed during early wood formation in *P. canariensis* [36], are overexpressed at H3, when late wood is already differentiating in other parts of the stem. On the contrary, other CAZymes (Contigs 01916, 17013 or 21865) or a cellulose synthase (Contig 15857), typically expressed during late wood formation in *P. canariensis* [36], are repressed at H2 and H3, in the same way as an early wood induced CCoAOMT (Contig 06476), crucial in lignin biosynthesis. Repression and overexpression of putative early and late wood genes during H1, H2 and H3 are summarized in Fig. 5.

**Fig. 6:**
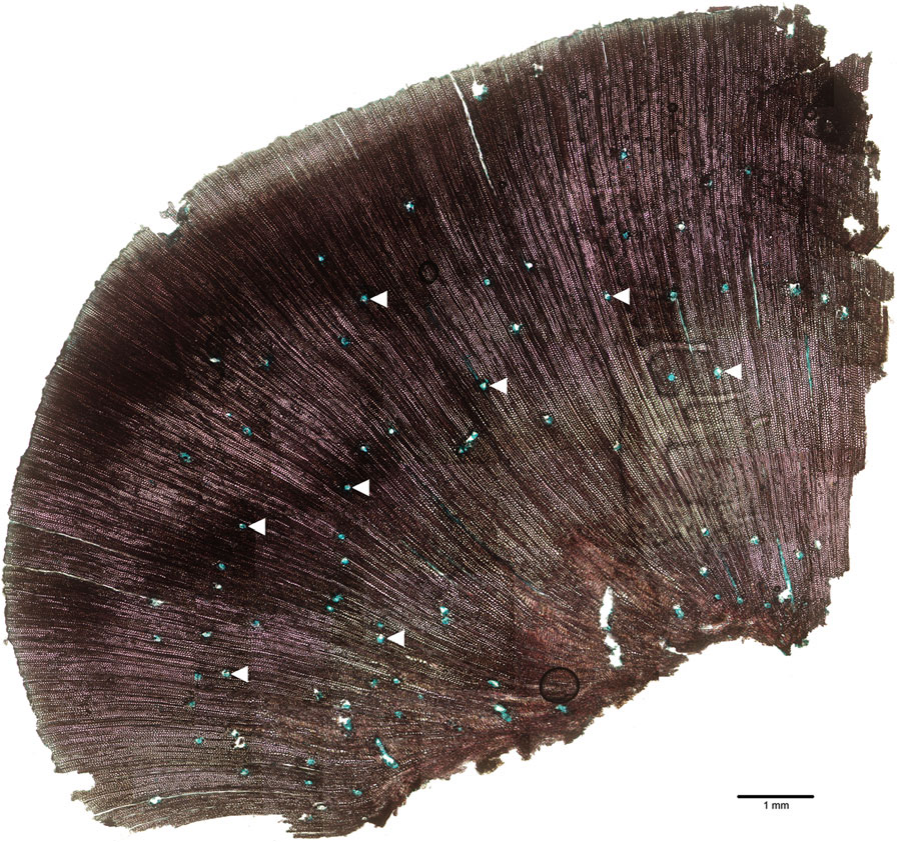
Micrograph of wound-wood. Bright-field microscopy picture of a 20 μm thick cross section of traumatic xylem 18 months after wounding

These results are consistent with anatomical observations. As shown in Fig. 6 not a clear difference between early and late wood is observed in the traumatic wood grown during 18 months after wounding. On the contrary, a high number of resin ducts appear in this traumatic wood, as already reported by Chano *et al* [9]. Accordingly, several genes related to resin synthesis have been detected as overexpressed at H2 and H3. **Oleoresins** are one of the main conifer defenses against pathogens, avoiding the spread of infections. In this work we have detected DEGs encoding geranyl diphosphate synthase and geranylgeranyl diphosphate synthase (Contig 03270 and Ppnisotig 10634, respectively; cluster H), involved in the synthesis of mono and diterpenes, induced at H3. In the same way, in the same cluster, overexpressed at H3, appears Contig 08417, encoding an abietadienol/abietadienal oxidase–like protein, which catalyzes several oxidative steps in diterpenol biosynthesis [78]. Contig 13499, encoding a (-)-camphene tricyclene synthase-like is also induced at H3, appearing in cluster C. This monoterpene synthase is involved in the synthesis of different monoterpenes, as camphene, tricyclene, limonene or myrcene [79].

Also induced at H2/H3 appear several genes presumably involved in ethylene synthesis, although this hormone is supposed to act in the first steps of the response [62]. This is the case of ACS (1-aminocyclopropane-1-carboxylic acid synthase) or ACO (1-aminocyclopropane-1-carboxilate oxidase). In spruce and Douglas fir, for instance, multiple ACS genes and a single ACO gene were found to be induced during the immediate response to wounding [80]. Conversely, we found multiple ACO genes overexpressed during the whole response (from H1 to H3), such as Contig 00524 (cluster C) and Contig 16100 (cluster I). This result suggests a different response in *P. canariensis* compared to *Picea* and *Pseudotsuga*, which could be related with the efficient healing response of Canary Island pine.

On the contrary, transcript levels of many other **defensive genes** overexpressed at H1 decrease to normal levels at H2/H3, or are even repressed at H3, when latewood is forming in other parts of the tree. During latewood formation defensive genes are expressed, as previously reported in *P. canariensis* [36] or *C. japonica* [37]. This constitutive upregulation of these genes could account for the comparatively lower expression levels detected for traumatic wood formation. This is the case of two HSPRO genes (Contigs 02906 and 19857), induced at H1 and related to nematode resistance, or a noduline (Contig 12353), presumably involved in plant-microbe interactions, which show lower transcription levels at H2/H3 in the healing borders than in controls.

As exposed previously for the immediate response, other non-annotated DEGs were significantly overexpressed at H2 and/or H3. Thus, 26 non-annotated DEGs were included in cluster C, and 80 more in cluster H. For instance, Contig 21346, with FC values over 7 in H2 and H3, or Contig 12627, with a FC value of 4.42 in H2 and 8.62 in H3). Important numbers of non-annotated sequences were found in clusters G and L, where 58 and 70 DEGs, respectively, showed underexpression for H2 and/or H3 (f.i., Contigs 20076, 23934 or 24690 in cluster G were strongly repressed at H3, with FC values close to -10, or Contigs 14134 and 20478, repressed for H2 and H3 with FC values from -9.5 to -3.12 and from -5.43 to -2.76, respectively). As well, some poorly annotated contigs were remarkably repressed. For instance, Contig 02798 (cluster L) and Contig 12685 (cluster G), homologous to uncharacterized LOC101210414, and LOC101213469 from *Cucumis sativus* [81], showed FC values of -5.06 and -6.43 at H3, respectively.

## Conclusions

Wounding induces a complete rearrangement of the transcriptional programme in the cambial zone close to the injuries. In particular, a considerable percentage of genes presumably involved in xylogenesis show an altered transcription pattern in response to wound and during healing.

At the first instance, radial growth is stopped in the vicinity of the wound, and a complete set of defensive genes, mostly related to biotic stress, are induced, as a barrier against opportunistic pathogens. Interestingly, some of these genes have also been reported to be preferentially transcribed in differentiating late wood. Later on, cambial activity is restored in the lateral borders of the wound, even at a higher rate than in other parts of the stem. This fast growth, which is dependent on the general health and reserves of the tree, eventually leads to the complete healing of the wound and restoration of the cambial ring. Anatomically, we have not detected a well-defined contrast among early and late wood in the traumatic wood formed during 18 months after wounding. During this period, most of the genes preferentially expressed during normal late wood development do not change their expression pattern, described in Chano *et al.* [36]. However, a subset of genes shows their transcription levels significantly altered by wound and healing. Among them, it is noteworthy the presence of genes involved in cell wall formation. Thus, genes coding for CAZymes and cellulose synthases overexpressed in normal late wood formation are comparatively repressed in traumatic wood. Conversely, similar genes typical of early wood keep their high transcription levels in traumatic wood, even at the moment of late wood formation. On the contrary, an early wood CCoAOMT, involved in lignin biosynthesis, is also repressed in traumatic wood. These genes, together with many others non-annotated yet, but showing similarly modified transcription patterns in healing tissue, probably underlie the anomalous characteristics of traumatic wood. In the same way, we cannot discard that other genes not detected as DEG due to the restrictive criteria used in this work could still play a biologically significant role in the wound wood formation process.

Our results suggest that the tree, after the synthesis of defensive molecules against eventual pathogens, and once cambial activity is restored at the wound borders, produces a fast growing traumatic wood. This tissue, in which annual rings are not clearly distinguished, at least the first year, could be less efficient as preventive barrier than normal late wood regarding secondary wall lignification, but it presents a high proportion of resin ducts, and also provides a good way to heal the wound in the shortest possible time. Further investigations are needed to clarify this point.

## Methods

### Plant material and wounding

**Fig. 7:**
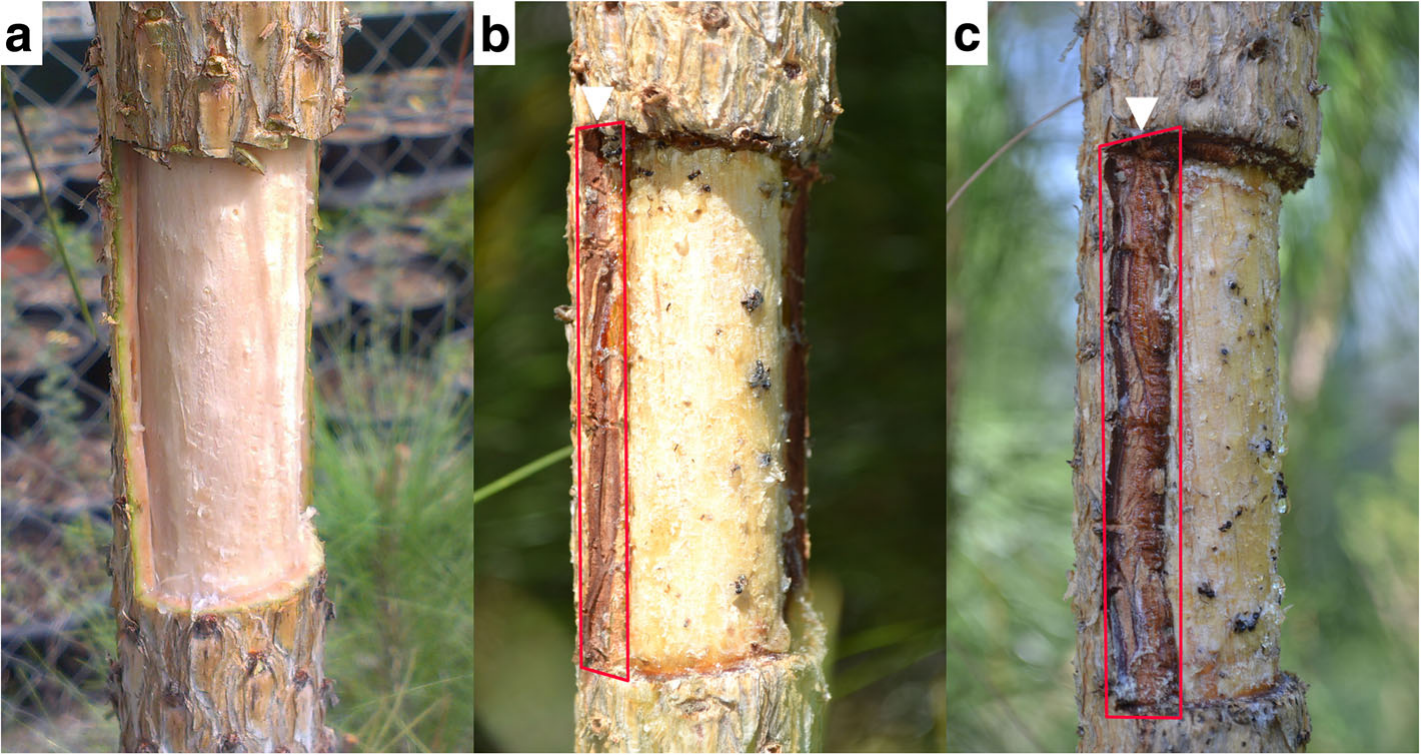
Wounded stem of *P. canariensis* at the sampling dates*.* **a** H1: 7 days after wounding; **b** H2: 78 days after wounding; **c**: 92 days after wounding

For this work we used 3 *Pinus canariensis* trees, 5 years old. Pines were grown in greenhouse, using 650 ml conical containers with 3:1 (v/v) peat:vermiculite. After the first year, trees were transferred to soil in experimental garden at UPM facilities, and grown under environmental conditions. At the moment of the beginning of this experiment, trees were approximately 2 m high and 7-10 cm diameter at the base. Using a sterile scalpel, we performed two wounds, removing bark, phloem, vascular cambium and first rows of xylem from a rectangular window 10 cm high and spanning half the circumference of the stems (Fig. 7). Wounds were performed in opposite sides of the stem and with an interval of approximately three wound heights.

Samples were collected according to the described seasonal growth and healing patterns described previously for the species [9, 36] Wounding was performed on April 9^th^, when cambial activity was ongoing. One week later we collected a frame of tissue from the wound margins in both wounds (H1); at this moment formation of a first traumatic tissue can be expected [9]. On June 25^th^, when the callous tissue was emerging from the margins of wounds and the trees were at the end of the early-wood development period, we collected the tissues growing in the margins of one wound per tree (H2). Later on, on July 9^th^, concurring with the late-wood development period [36], we collected the thick callous tissues growing in the frame of remaining wounds (H3).

For each sample, controls were collected at the same sampling dates, from branches away from the wounds, in order to distinguish transcriptomic changes of the vegetative growth of those caused by wounding. The tissue collected for control samples included bark, phloem, vascular cambium and the most external layers of xylem. Collected samples were processed individually, immediately frozen in liquid nitrogen and stored a -80°C.

### RNA isolation and sequencing

Total RNA was isolated from each sample, using the CTAB-LiCl precipitation method [82], and purified with the RNeasy Plant Mini Kit (Qiagen, CA, USA). Quantity of total RNA for each sample was measured with Nanodrop model ND-1000 (Thermo Scientific, MA, USA), and RNA quality was checked using Experion Bioanalyzer (Bio-Rad, CA, USA).

### Microarray analysis

A set of 15266 contigs involved in meristematic activity of *Pinus canariensis*, selected from a previous work [36], was used for the design of a two-color 60K microarray (Agilent, USA). Furthermore, we added 2303 contigs from other cDNA libraries of *P. pinea*, as well ESTs and sequences of the loblolly pine from the Pine Gene Index Database (http://www.mgel.msstate.edu/dna_libs.htm). For each contig, one 60 bp long probe was designed and spotted at least 3 times on the slide. Probes designed for *Populus*, mouse and human ESTs available in public databases were included as negative controls.

For each sampling point (H1, H2, H3), the three biological replicates were hybridized (wound vs. control) following the two-color protocol provided by the manufacturer (Agilent Technologies, CA, USA), and images were captured with a GenePix 4000B (Axon, CA, USA), and spots were quantified using the GenePix software (Axon, CA, USA). Microarray data was uploaded to the NCBI’ Gene Expression Omnibus and are accessible through the GEO series accession number GSE102275 (https://www.ncbi.nlm.nih.gov/geo/query/acc.cgi?acc=GSE102275).

Background correction and normalization of expression data were performed using LIMMA (Linear Models for Microarray Data; [83]). For local background correction and normalization, the methods “normexp” and “loess” in LIMMA were used, respectively. To achieve similar distribution across arrays and consistency among arrays, log-ratio values were scaled using the median-absolute value as scale estimator.

The non-parametric algorithm “Rank Products”, available as a package for Bioconductor in R [84–86], was used for evaluation of Differentially Expressed Genes (DEGs). This method detects genes that are consistently high ranked in a number of replicated experiments independently of their numerical intensities. Results are provided in the form of *P* adjusted by False Discovery Rate (FDR), defined as the probability of a given gene is ranked in the observed position by chance.

Those probes with a FC above 2 and below -2, with a significance level FDR below 0.05, were selected as differentially expressed. Thus, technical replicates were merged into one value per contig, and a datamatrix formed by ratios between experimental and control measurements for selected Differentially Expressed Genes (DEGs), including time sampled and biological replicate, was created. Clustering was performed in R, and the heatmap was plotted using the heatmap.2 function of the gplots package [87]. Enrichment analysis of DEGs was performed using Blast2GO v.2.7.2 as well.

### qRT-PCR validation

The expression patterns of 12 DEGs covering the main profiles obtained from microarrays were confirmed by qRT-PCR using the same RNA employed for microarray hybridizations. First strand cDNA synthesis was performed using SuperScript™ III reverse transcriptase (Invitrogen, USA) following manufacturer’s instructions and using 4 μgr of total RNA and random hexamers. Gene specific primers were designed for twelve selected DEGs (Table 2) using the Primer3 software [88], with a melting temperature between 60 and 65° C, and producing amplicons between 80 and 120 bp. qRT-PCR was performed in a CFX96™ Real-Time PCR Detection System (Biorad, USA), using the SsoFast™ EVAgreen® Supermix (Biorad, USA), according to manufacturer’s protocol, and following the standard thermal profile: 95° C for 3 min, 40 cycles of 95° C for 10 s and 60° C for 10 s. In order to compare data from different qRT-PCR runs, the CT values were normalized using the Ri18S as housekeeping gene, whose specific primers were FW 5’-GCGAAAGCATTTGCCAAGG-3’ and REV 5’-ATTCCTGGTCGGCATCGTTTA-3’. This genes has been previously proved to be useful for this purpose in pine species (f.i., see Perdiguero et al. [89]). The expression ratios were then obtained using the delta-delta-CT method corrected for the PCR efficiency for each DEG [90].

**Table 2:** Primers used for qRT-PCR

Contig name	Oligo name	Description		bp	Tm	GC%	Sequence (5’-3’)
Contig00654	Pc_00654_CESA_F1	cellulose synthase a-like protein	Forward	20	63.0	55	GGACCACACTCCTCATTCCT
	Pc_00654_CESA_R1		Reverse	20	63.0	45	ACCCCATGACTGAAATCCAT
Contig12050	Pc_12050_MYB_F1	MYB46-like protein	Forward	20	62.8	45	ATTCCCAACATGGAAGAAGC
	Pc_12050_MYB_R1		Reverse	20	63.7	50	CTGCATCACCATCACACTCA
Contig20304	Pc_20304_ATHB13_F1	ATHB13-like protein	Forward	20	63.2	50	CCCATTCTCATGATGTCTGC
	Pc_20304_ATHB13_R1		Reverse	20	63.1	50	CAGAACTGCCTTCACTTCCA
Contig00787	Pc_00787_NAC_F1	NAC2-like prtoein	Forward	20	62.5	45	CTAAATGGCCCTGGGTAAAA
	Pc_00787_NAC_R1		Reverse	20	62.8	50	CCCCTTCTTCTTACCAACCA
Contig20555	Pc_20555_PAL_F1	phenylalanine ammonia-lyase-like protein	Forward	20	63.1	50	GAATTGACGTCCTGGTTGTG
	Pc_20555_PAL_R1		Reverse	20	62.7	50	CAGCCTGGACTATGGTTTCA
Contig03225	Pc_03225_EXPANSIN_F1	α-expansin-like protein	Forward	20	62.8	45	AAGCGGAGCTGATTCTTGAT
	Pc_03225_EXPANSIN_R1		Reverse	20	63.1	60	CTCAGAGCCACAGAGACGAG
Contig05551	Pc_05551_WRKY_F1	WRKY51-like protein	Forward	20	62.5	45	ACGCAGAGGGGAATAAGAAA
	Pc_05551_WRKY_R1		Reverse	20	63.2	50	CAGAAAACGTTCACCCACAG
Contig06476	Pc_06476_CCoAOMT_F1	CCoAOMT-like protein	Forward	20	64.0	50	GATTGAACAACCGAGGTGCT
	Pc_06476_CCoAOMT_R1		Reverse	20	63.6	45	TGCAACACCTGAATTCCAAC
Contig06813	Pc_06813_WOX_F1	WOX4-like protein	Forward	20	63.1	50	TCTCGGCTCATGTTCACTTC
	Pc_06813_WOX_R1		Reverse	20	63.1	50	TACCAGTGGTTGCAGGTGTT
Contig09007	Pc_09007_EXO_F1	exordium 2-like protein	Forward	20	62.9	45	TACCCGATCATGCAAGACAT
	Pc_09007_EXO_R1		Reverse	20	62.7	55	GCGCCTAAATCTACCTGCTC
Contig05923	Pc_05923_bHLH_F1	bHLH35-like protein	Forward	20	63.9	45	GTGCGAATAGAGGGCAAAAA
	Pc_05923_bHLH_R1		Reverse	20	64.1	45	CGAAGCAGCAGATGTTTGAA
Contig22185	Pc_22185_PR_F1	Major allergen PRU-like protein	Forward	20	65.0	60	GTGGAGGCAAGGAGACTGTG
	Pc_22185_PR_R1		Reverse	19	64.9	63.2	CTGCCTACGCCTCCATCTC
House-keeping	Ri18S_FW	18S ribosomal	Forward	19	62.4	53	GCGAAAGCATTTGCCAAGG
	Ri18S_RV		Reverse	21	62.4	48	ATTCCTGGTCGGCATCGTTTA

Tm: Melting temperature. GC%: guanine-cytosine content

## Additional files

Additional file 1: Wound-responsive differentially expressed genes. FC: fold change. FDR: adjusted p-value by False Discovery Rate. In bold, statistically significant values. (XLSX 151 kb)

Additional file 2: Hierarchical clustering of samples. (PNG 22 kb)
