# Supplementary figures and images for: Transcriptomic analysis of wound xylem formation in Pinus canariensis
Source: BMC Plant Biol. 2017 Dec 4;17:234. doi: 10.1186/s12870-017-1183-3 (PMC5715621; doi:10.1186/s12870-017-1183-3)

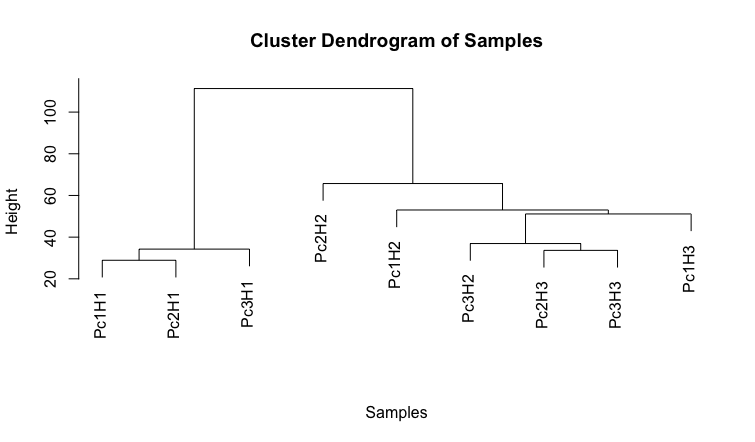

Supplement: Supplementary file 2 — Hierarchical clustering of samples. (PNG 22 kb) [file 12870_2017_1183_MOESM2_ESM.png]
